# Methyl 5-bromo-2-hy­droxy­benzoate

**DOI:** 10.1107/S1600536812016297

**Published:** 2012-04-21

**Authors:** Ghulam Mustafa, Islam Ullah Khan, Muhammad Zar Ashiq, Mehmet Akkurt

**Affiliations:** a Department of Chemistry, GC University, Lahore 54000, Pakistan; b National Center of Excellence in Physical Chemistry, University of Peshawar, Peshawar 25000, Pakistan; c Department of Physics, Faculty of Sciences, Erciyes University, 38039 Kayseri, Turkey

## Abstract

The title compound, C_8_H_7_BrO_3_, is almost planar (r.m.s. deviation for the non-H atoms = 0.055 Å). In the crystal, O—H⋯O hydrogen bonds link the mol­ecules into *C*(6) chains propagating in [010]. Very weak aromatic π–π inter­actions [centroid–centroid distances = 3.984 (5) and 3.982 (5) Å] also occur.

## Related literature

For the crystal structure of the methyl 4-bromo-3-hy­droxy­benzoate isomer, see: Huang *et al.* (2011 ▶). For graph-set notation, see: Bernstein *et al.* (1995 ▶).
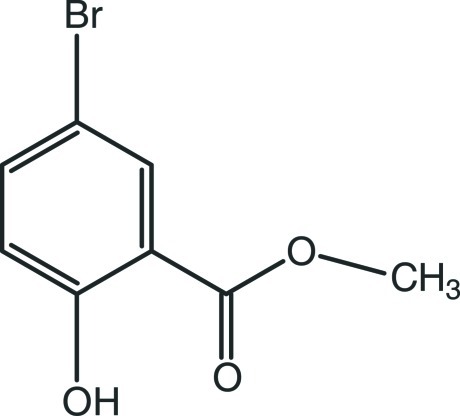


## Experimental

### 

#### Crystal data

C_8_H_7_BrO_3_
*M* *_r_* = 231.04
Monoclinic, 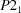

*a* = 3.9829 (8) Å
*b* = 9.0950 (19) Å
*c* = 12.122 (3) Å
β = 95.162 (9)°
*V* = 437.33 (17) Å^3^
*Z* = 2
Mo *K*α radiation
μ = 4.66 mm^−1^
*T* = 296 K
0.34 × 0.28 × 0.23 mm

#### Data collection

Bruker APEXII CCD diffractometer
Absorption correction: multi-scan (*SADABS*; Bruker, 2005 ▶) *T*_min_ = 0.228, *T*_max_ = 0.342
3242 measured reflections
1644 independent reflections
1186 reflections with *I* > 2σ(*I*)
*R*_int_ = 0.057

#### Refinement

*R*[*F* ^2^ > 2σ(*F* ^2^)] = 0.058
*wR*(*F* ^2^) = 0.142
*S* = 1.06
1644 reflections
112 parameters
1 restraint
H-atom parameters constrained
Δρ_max_ = 1.31 e Å^−3^
Δρ_min_ = −0.72 e Å^−3^
Absolute structure: Flack (1983 ▶), 687 Freidel pairs
Flack parameter: 0.07 (3)

### 

Data collection: *APEX2* (Bruker, 2007 ▶); cell refinement: *SAINT* (Bruker, 2007 ▶); data reduction: *SAINT*; program(s) used to solve structure: *SHELXS97* (Sheldrick, 2008 ▶); program(s) used to refine structure: *SHELXL97* (Sheldrick, 2008 ▶); molecular graphics: *ORTEP-3* (Farrugia, 1997 ▶) and *PLATON* (Spek, 2009 ▶); software used to prepare material for publication: *WinGX* (Farrugia, 1999 ▶) and *PLATON*.

## Supplementary Material

Crystal structure: contains datablock(s) global, I. DOI: 10.1107/S1600536812016297/hb6740sup1.cif

Structure factors: contains datablock(s) I. DOI: 10.1107/S1600536812016297/hb6740Isup2.hkl

Supplementary material file. DOI: 10.1107/S1600536812016297/hb6740Isup3.cml

Additional supplementary materials:  crystallographic information; 3D view; checkCIF report

## Figures and Tables

**Table 1:** Hydrogen-bond geometry (Å, °)

*D*—H⋯*A*	*D*—H	H⋯*A*	*D*⋯*A*	*D*—H⋯*A*
O1—H1⋯O2^i^	0.82	2.25	3.065 (10)	170

Symmetry code: (i) 
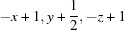
.

**Fig. 1.**
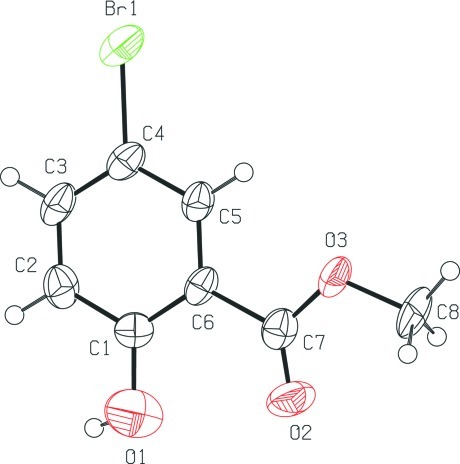
The molecular structure of the title compound with displacement ellipsoids for non-H atoms drawn at the 50% probability level.

**Fig. 2.**
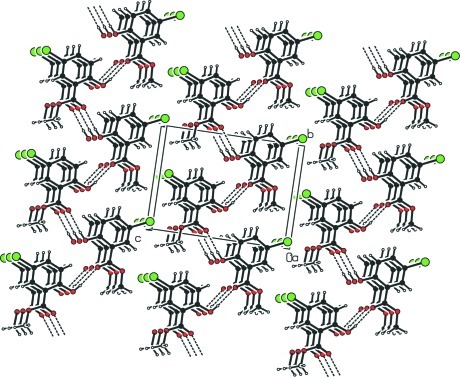
View of the packing and hydrogen-bonding (dotted lines) of the title compound along the a axis.

## supplementary crystallographic information

### Comment

In the title compound (I), (Fig. 1), all bond lengths and angles are comparable with those of its isomer methyl 4-bromo-3-hydroxybenzoate (Huang *et al.*, 2011). These isomers crystallize in the monoclinic *P* 2_1_ (*Z*= 2) and *P* 2_1_/c (*Z*=4) space groups, respectively.

Both these crystals have two different supramolecular O—H···O hydrogen-bond patterns. In the crystal, molecules are linked by O—H···O hydrogen bonds (Table 1), forming a zigzag chain of C(6) motifs (Bernstein *et al.*, 1995) along the [010] and are further interlinked through very weak π-π stacking interactions [centroid-centroid distances = 3.984 (5) and 3.982 (5) Å] between the benzene rings, along the [1 0 0] axis (Table 1 and Fig. 2).

### Experimental

The title compound was prepared by dissolving methy-5-bromo-2-hydroxybenzoic acid (1.0 g, 4.6 mmol) in DMF (10 ml) and n-hexane washed sodium hydride (0.22 g, 9.0 mmol). The whole mixture was astirred at room temperature for 45 min followed by the addition of methyl iodide (0.85 g, 5.9 mmol). The whole reaction mixture was stirred at room temprature till the completion of the reaction and poured into crushed ice in a beaker. The pH of the mixture was adjusted to 4.0 with 1 N HCl. Precipitates were produced, filtered and washed twice with distilled water and crystallized from chloroform solution as yellow-brown needles.

### Refinement

All H atoms were positioned with idealized geometry and were refined using a riding model with *U*_iso_(H) = 1.2 or 1.5*U*_eq_(C) [O—H = 0.82 Å, C—H = 0.93 and 0.96 Å]. Four poorly fitted reflections (0 - 1 1), (-1 0 10), (0 1 1) and (1 6 3) were omitted from the refinement.

### Crystal data

**Table:**

C_8_H_7_BrO_3_	*F*(000) = 228
*M**_r_* = 231.04	*D*_x_ = 1.755 Mg m^−^^3^
Monoclinic, *P*2_1_	Mo *K*α radiation, λ = 0.71073 Å
Hall symbol: P 2yb	Cell parameters from 1516 reflections
*a* = 3.9829 (8) Å	θ = 2.8–24.2°
*b* = 9.0950 (19) Å	µ = 4.66 mm^−^^1^
*c* = 12.122 (3) Å	*T* = 296 K
β = 95.162 (9)°	Needle, yellow–brown
*V* = 437.33 (17) Å^3^	0.34 × 0.28 × 0.23 mm
*Z* = 2	

### Data collection

**Table:**

Bruker APEXII CCD diffractometer	1644 independent reflections
Radiation source: sealed tube	1186 reflections with *I* > 2σ(*I*)
Graphite monochromator	*R*_int_ = 0.057
φ and ω scans	θ_max_ = 26.5°, θ_min_ = 3.4°
Absorption correction: multi-scan (*SADABS*; Bruker, 2005)	*h* = −4→4
*T*_min_ = 0.228, *T*_max_ = 0.342	*k* = −11→11
3242 measured reflections	*l* = −15→15

### Refinement

**Table:**

Refinement on *F*^2^	Secondary atom site location: difference Fourier map
Least-squares matrix: full	Hydrogen site location: inferred from neighbouring sites
*R*[*F*^2^ > 2σ(*F*^2^)] = 0.058	H-atom parameters constrained
*wR*(*F*^2^) = 0.142	*w* = 1/[σ^2^(*F*_o_^2^) + (0.0687*P*)^2^] where *P* = (*F*_o_^2^ + 2*F*_c_^2^)/3
*S* = 1.06	(Δ/σ)_max_ < 0.001
1644 reflections	Δρ_max_ = 1.31 e Å^−^^3^
112 parameters	Δρ_min_ = −0.72 e Å^−^^3^
1 restraint	Absolute structure: Flack (1983), 687 Freidel pairs
Primary atom site location: structure-invariant direct methods	Flack parameter: 0.07 (3)

### Special details

**Table:**

Geometry. Bond distances, angles *etc*. have been calculated using the rounded fractional coordinates. All su's are estimated from the variances of the (full) variance-covariance matrix. The cell e.s.d.'s are taken into account in the estimation of distances, angles and torsion angles
Refinement. Refinement on *F*^2^ for ALL reflections except those flagged by the user for potential systematic errors. Weighted *R*-factors *wR* and all goodnesses of fit *S* are based on *F*^2^, conventional *R*-factors *R* are based on *F*, with *F* set to zero for negative *F*^2^. The observed criterion of *F*^2^ > σ(*F*^2^) is used only for calculating -*R*-factor-obs *etc*. and is not relevant to the choice of reflections for refinement. *R*-factors based on *F*^2^ are statistically about twice as large as those based on *F*, and *R*-factors based on ALL data will be even larger.

### Fractional atomic coordinates and isotropic or equivalent isotropic displacement parameters (Å^2^)

**Table:**

	*x*	*y*	*z*	*U*_iso_*/*U*_eq_	
Br1	1.1035 (2)	1.05024 (13)	0.06707 (7)	0.0606 (3)	
O1	0.663 (2)	0.7888 (8)	0.4895 (6)	0.076 (3)	
O2	0.4780 (15)	0.5536 (9)	0.3653 (4)	0.0601 (18)	
O3	0.6181 (14)	0.5492 (9)	0.1924 (4)	0.0512 (18)	
C1	0.759 (2)	0.8434 (8)	0.3920 (6)	0.035 (3)	
C2	0.901 (2)	0.9860 (8)	0.3906 (7)	0.043 (3)	
C3	0.9992 (16)	1.0454 (12)	0.2961 (6)	0.042 (2)	
C4	0.966 (2)	0.9673 (8)	0.1995 (7)	0.039 (3)	
C5	0.8380 (19)	0.8249 (8)	0.1976 (6)	0.037 (3)	
C6	0.736 (2)	0.7643 (7)	0.2943 (6)	0.033 (2)	
C7	0.594 (2)	0.6113 (8)	0.2910 (7)	0.039 (3)	
C8	0.478 (3)	0.4012 (8)	0.1801 (8)	0.060 (4)	
H1	0.64600	0.85650	0.53340	0.1140*	
H2	0.92750	1.04020	0.45590	0.0520*	
H3	1.08960	1.13970	0.29710	0.0510*	
H5	0.82060	0.77090	0.13220	0.0440*	
H8A	0.59580	0.33700	0.23330	0.0910*	
H8B	0.50350	0.36590	0.10670	0.0910*	
H8C	0.24340	0.40340	0.19220	0.0910*	

### Atomic displacement parameters (Å^2^)

**Table:**

	*U*^11^	*U*^22^	*U*^33^	*U*^12^	*U*^13^	*U*^23^
Br1	0.0692 (6)	0.0456 (4)	0.0687 (6)	−0.0185 (6)	0.0153 (4)	0.0107 (5)
O1	0.105 (6)	0.059 (4)	0.066 (4)	0.024 (4)	0.019 (4)	0.002 (3)
O2	0.096 (4)	0.034 (2)	0.054 (3)	−0.004 (5)	0.027 (3)	0.009 (4)
O3	0.070 (4)	0.030 (2)	0.055 (3)	−0.022 (4)	0.014 (2)	−0.006 (4)
C1	0.035 (5)	0.031 (4)	0.038 (4)	0.009 (3)	0.001 (3)	0.006 (3)
C2	0.051 (5)	0.031 (4)	0.047 (5)	0.005 (4)	−0.001 (4)	−0.009 (3)
C3	0.040 (4)	0.024 (3)	0.061 (5)	−0.004 (5)	−0.003 (3)	0.007 (6)
C4	0.035 (4)	0.029 (4)	0.052 (5)	0.000 (3)	0.003 (4)	0.009 (3)
C5	0.039 (5)	0.024 (3)	0.046 (5)	−0.001 (3)	−0.001 (3)	−0.001 (3)
C6	0.031 (4)	0.019 (3)	0.049 (5)	0.002 (3)	0.001 (3)	0.004 (3)
C7	0.039 (5)	0.028 (3)	0.050 (5)	0.003 (3)	−0.003 (4)	0.003 (4)
C8	0.078 (7)	0.016 (4)	0.087 (7)	−0.015 (4)	0.008 (5)	−0.005 (4)

### Geometric parameters (Å, º)

**Table:**

Br1—C4	1.899 (8)	C4—C5	1.391 (10)
O1—C1	1.368 (10)	C5—C6	1.389 (10)
O2—C7	1.173 (10)	C6—C7	1.501 (10)
O3—C7	1.333 (10)	C2—H2	0.9300
O3—C8	1.460 (11)	C3—H3	0.9300
O1—H1	0.8200	C5—H5	0.9300
C1—C6	1.382 (10)	C8—H8A	0.9600
C1—C2	1.416 (10)	C8—H8B	0.9600
C2—C3	1.356 (11)	C8—H8C	0.9600
C3—C4	1.366 (12)		
			
C7—O3—C8	115.1 (7)	O3—C7—C6	111.1 (7)
C1—O1—H1	109.00	O2—C7—O3	124.3 (8)
O1—C1—C6	123.3 (7)	C1—C2—H2	119.00
C2—C1—C6	117.6 (7)	C3—C2—H2	119.00
O1—C1—C2	119.1 (7)	C2—C3—H3	120.00
C1—C2—C3	121.4 (8)	C4—C3—H3	120.00
C2—C3—C4	120.3 (9)	C4—C5—H5	120.00
Br1—C4—C5	119.3 (6)	C6—C5—H5	120.00
C3—C4—C5	120.4 (8)	O3—C8—H8A	109.00
Br1—C4—C3	120.4 (6)	O3—C8—H8B	109.00
C4—C5—C6	119.3 (7)	O3—C8—H8C	110.00
C1—C6—C7	120.1 (7)	H8A—C8—H8B	109.00
C5—C6—C7	118.9 (7)	H8A—C8—H8C	110.00
C1—C6—C5	121.0 (6)	H8B—C8—H8C	110.00
O2—C7—C6	124.6 (8)		
			
C8—O3—C7—C6	−178.4 (7)	C2—C3—C4—C5	−1.4 (12)
C8—O3—C7—O2	1.8 (12)	Br1—C4—C5—C6	−179.7 (6)
C6—C1—C2—C3	2.5 (12)	C3—C4—C5—C6	1.7 (12)
O1—C1—C2—C3	−179.8 (8)	C4—C5—C6—C7	179.2 (7)
C2—C1—C6—C5	−2.2 (12)	C4—C5—C6—C1	0.2 (12)
C2—C1—C6—C7	178.8 (7)	C1—C6—C7—O2	4.8 (13)
O1—C1—C6—C5	−179.8 (8)	C5—C6—C7—O3	6.0 (10)
O1—C1—C6—C7	1.2 (12)	C1—C6—C7—O3	−174.9 (7)
C1—C2—C3—C4	−0.7 (12)	C5—C6—C7—O2	−174.2 (8)
C2—C3—C4—Br1	−180.0 (6)		

### Hydrogen-bond geometry (Å, º)

**Table:**

*D*—H···*A*	*D*—H	H···*A*	*D*···*A*	*D*—H···*A*
O1—H1···O2^i^	0.82	2.25	3.065 (10)	170

Symmetry code: (i) −*x*+1, *y*+1/2, −*z*+1.
